# Exclusive human milk feeding and prevalence of early adiposity rebound in ELBW infants: a retrospective cohort study

**DOI:** 10.1007/s00431-023-05374-6

**Published:** 2023-12-19

**Authors:** Jacopo Cerasani, Alessandra Consales, Silvana Gangi, Marta Macchi, Daniela Morniroli, Giulia Vizzari, Valentina Tiraferri, Angelo Petrelli, Fabio Mosca, Maria Lorella Giannì

**Affiliations:** 1 Department of Clinical Sciences and Community Health, University of Milan, Milan, Italy; 2 NICU, Fondazione IRCCS Ca’ Granda Ospedale Maggiore Policlinico, Milan, Italy

**Keywords:** ELBW infants, Early adiposity rebound, Human milk, Metabolic outcomes

## Abstract

The purpose of the present study was to evaluate the prevalence of early adiposity rebound (EAR) and factors associated with its occurrence in a cohort of extremely low birth weight infants (ELBW). We conducted a retrospective longitudinal study including ELBW infants followed-up for up to 10 years after discharge. EAR was defined as occurring before 5 years of age. A multivariate binary logistic regression analysis was performed to evaluate maternal and perinatal variables independently associated with EAR. Out of 212 ELBW infants included in the analysis, 40.6% developed EAR and 21.5% showed it before 4 years of age. Only formula milk feeding at discharge was independently associated with a higher risk of EAR. The mean BMI of children with EAR was higher than that of children without EAR. Furthermore, the prevalence of overweight and obesity was higher in the EAR group than in the timely AR group.

*  Conclusions*: ELBW infants in our cohort developed EAR in a relatively high percentage of cases. In this already at-risk population, EAR may represent a further risk factor for an adverse metabolic outcome. Monitoring preterm infants’ growth within a long-term follow-up program and promoting and supporting human milk feeding is advisable.

**Table:** 

**What is Known:** *• Preterm-born infants are at high risk for long-term adverse health outcomes, especially cardiovascular and metabolic.* *• The occurrence of early adiposity rebound (EAR) is associated with the risk of later obesity and metabolic syndrome.*	
**What is New:** *• The occurrence of EAR in ELBW infants may represent an additional risk factor for later adverse metabolic outcomes in an already vulnerable population.* *• Future preventive strategies should include a long-term follow-up and the promotion of exclusive breastfeeding.*

**Supplementary Information:**

The online version contains supplementary material available at 10.1007/s00431-023-05374-6.

## Introduction

Preterm birth is a worldwide epidemic. Approximately 15 million preterm infants are born each year, and prematurity-related complications still represent one of the most common causes of death during the first years of life [1, 2]. However, due to the improvements in neonatal assistance, the survival rate of preterm infants continues to increase, particularly among extremely preterm infants, allowing an ever-growing number of them to reach adulthood [3].

Increasing evidence indicates that survivors of premature birth are at high risk for adverse health outcomes later in life, such as cardiovascular and metabolic diseases, due to the early interruption of organ maturation, and subsequent altered structure and function. Remarkably, this risk increases as gestational age at birth decreases [4]. Several perinatal events, including comorbidities associated with prematurity, could exacerbate the increased susceptibility of preterm infants by inducing permanent adaptative changes in organ development through epigenetic mechanisms, and structural damage caused by the pro-inflammatory response and abnormal tissue repair [5].

The growth pattern has been reported to play a crucial role in modulating the risk of long-term health consequences of preterm infants in adult age [4, 6]. Indeed, the accelerated postnatal growth most preterm infants undergo to recover the extrauterine growth retardation developed during hospital stay imposes a metabolic cost in terms of altered body composition development and metabolic biomarkers [7].

Routine monitoring of body mass index (BMI) may aid the early detection of infants at risk for adverse metabolic outcomes. BMI rapidly increases during the first year of life, then progressively decreases and eventually starts rising again between the ages of 5 and 7. This second rise is called adiposity rebound (AR). An AR occurring at a younger age is defined early adiposity rebound (EAR). An association between EAR and the risk of later obesity and metabolic syndrome has been reported by several Authors [8–10].

Data on the prevalence of EAR in preterm infants and the factors associated with its occurrence are limited [11, 12], and no study so far has focused specifically on the evaluation of the BMI trajectory of infants born with extremely low birth weight (ELBW; i.e., birth weight < 1000 g).

The present study aimed to evaluate the prevalence of EAR and the factors associated with its occurrence in a cohort of ELBW infants. We also aimed to compare the rate of overweight and obesity among children in the EAR and in the timely AR group, as well as their blood pressure values from 4 years of age onward.

## Methods

### Study design

We conducted a retrospective longitudinal observational study including ELBW infants born between 2008 and 2016 admitted to the neonatal intensive care unit (NICU) of our Institution and participating in the 10-year follow-up program routinely provided after discharge to all inborn very preterm infants. Patients were selected from a prospectively filled database by 2 experienced neonatologists. Patients with genetic syndromes or endocrinological issues potentially affecting growth were excluded from the analysis.

The Ethics Committee of the Fondazione IRCCS Cà Granda Ospedale Maggiore Policlinico, Milan (Italy) approved the study. We obtained parental consent to use anonymized data for research purposes. The study was conducted in accordance with the Declaration of Helsinki.

### Data collection

Maternal and neonatal clinical data were collected from the patients’ computerized medical charts (Neocare, i&t Informatica e Tecnologia Srl, Italy). Maternal data included the following: age, educational level, single or multiple pregnancy, mode of delivery, and the occurrence of preeclampsia. Neonatal data included the following: gender, gestational age, Apgar score, birth weight, length, and head circumference at birth. Based on the birth weight percentile, infants were classified as adequate for gestational age (AGA; i.e., birth weight between 10th–90th centile), small for gestational age (SGA; i.e., birth weight < 10th centile) and large for gestational age (LGA; i.e., birth weight > 90th centile) according to the Intergrowth-21st charts [13]. Furthermore, days of non-invasive or invasive ventilation, the occurrence of comorbidities during hospital stay (i.e., severe brain and surgical gastrointestinal diseases, bronchopulmonary dysplasia—BPD, defined according to Jobe and Bancalari’s definition [14]—retinopathy of prematurity—ROP [15], infections), length of hospital stay, and mode of feeding at discharge (exclusive human milk, mixed feeding, exclusive formula milk) were also collected.

Anthropometric parameters (i.e., weight, length/height, and head circumference) measured after discharge at follow-up visits at 1 and 2 years (corrected age) and at 3, 4, 5, 6, 7, 8.5, and 10 years (chronological age) were collected from computerized medical charts. Body weight and length/height were measured according to standard procedures [16]. For each measurement, the appropriate centile was calculated according to the Centers for Disease Control and Prevention Growth Charts until 2 years corrected age and the Cacciari Growth Charts from the second year of age [17, 18]. Catch-up growth was defined as the achievement of a weight or length ≥ 10th centile [19, 20]. From the age of 2 years, we calculated the BMI expressed as weight in kilograms divided by height in meters squared. In line with the current literature, we defined the timing of AR as the age at the lowest BMI registered [10]. If AR occurred before 5 years of age, we classified it as EAR. According to the BMI growth charts of the World Health Organization (WHO), we classified children with a BMI ≥ 85th percentile as overweight and those with a BMI ≥ 97th as obese [21]. Finally, from the age of 4 years, as for Institutional protocol, the values of systolic and diastolic arterial blood pressure (SP and DP) were recorded at each outpatient visit.

### Statistical analysis

Statistical analysis was performed using SPSS (Statistical Package for the Social Sciences) statistic software package (IBM SPSS Statistics for Windows, Version 25.0. Armonk, NY: IBM Corp.). Continuous variables were expressed as mean values and standard deviations (SD), while categorical variables were presented as frequencies and percentages.

Values of BMI and arterial blood pressure (SP and DP) were compared between children with or without EAR using the Student’s *t*-test. The difference in proportions of overweight and obesity among children with EAR and children with timely AR was assessed using the chi-square test.

A multivariate binary logistic regression analysis was performed to identify variables independently associated with EAR. The variables included in the model were: maternal educational level, single or multiple pregnancy, mode of delivery, gender, being born SGA, and type of feeding at discharge. The selection of variables to include in the model was guided by their known or suspected associations with adiposity based on existing literature [22–26] and clinical relevance.

A Repeated Measures ANOVA was performed to evaluate the effect of EAR on BMI measured at different time points.

For all tests, a *p* value < 0.05 was considered statistically significant.

## Results

**Fig. 1:**
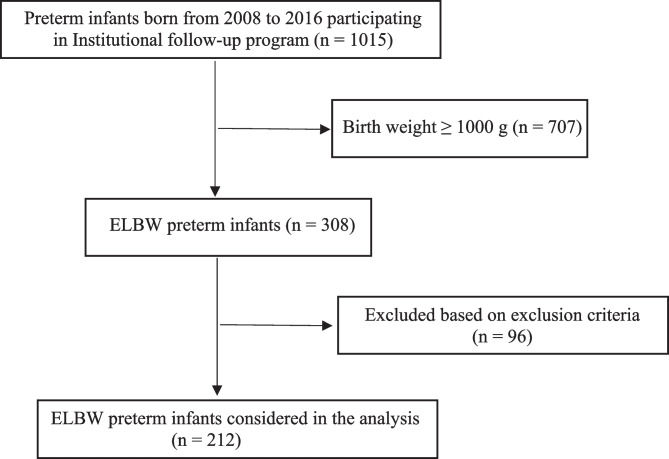
Flow-chart of the study

From 2008 to 2016, a total of 69,069 newborns were born at our Institution. Among them, 1015 preterm patients participated in the follow-up program provided at our Institution. A total of 212 ELBW infants were included in the study, while 803 patients did not meet the inclusion criteria or were excluded based on the exclusion criteria (Fig. 1). The 70% of the enrolled infants reached the 7th-year follow-up visit, while the 24% completed the 10-year follow-up program.

Maternal and neonatal characteristics are summarized in Table 1. Mean maternal age was 34.8 years, and 47.9% of mothers had a University degree. Nearly 38% of pregnancies were multiple and preeclampsia complicated one third of the total. Mean gestational age was 27 weeks, while mean weight, length, and head circumference at birth were 802 g, 33 cm, and 24 cm, respectively. Nearly half of the infants were SGA. The 14% of the infants enrolled were exclusively breastfed at discharge.

**Table 1:** Pregnancy-related and neonatal characteristics of the study population, divided by occurrence of early adiposity rebound (EAR)

Variable	Total Population (*n* = 212)	EAR (*n* = 86)	Timely AR (*n* = 126)	*p*
**Pregnancy**				
Single, *N* (%)	132 (62.3)	53 (61.6)	79 (62.7)	0.82
Multiple, *N* (%)	80 (37.7)	33 (38.4)	47 (37.3)	
**Preeclampsia**	70 (33.0)	33 (38.4)	37 (29.3)	0.14
**Mode of delivery**				
Vaginal delivery	28 (13.2)	11 (12.8)	17 (13.5)	0.88
Caesarean section	184 (86.8)	75 (87.2)	109 (86.5)	
**Newborn gender**				
Male	90 (42.4)	38 (44.2)	52 (41.3)	0.74
Female	122 (57.5)	48 (55.8)	74 (58.7)	
**Gestational age (weeks), mean (SD)**	27.1 (2.2)	27.0 (2.2)	27.1 (2.1)	0.75
**5′ Apgar score, mean (SD)**	7.9 (1.0)	7.9 (1.0)	7.9 (1.1)	0.95
**Birth weight (g), mean (SD)**	802.7 (138.3)	819 (129)	792 (144)	0.20
**AGA, *N* (%)**	117 (55.1)	50 (58.1)	67 (53.2)	0.68
**SGA, *N* (%)**	91 (42.9)	34 (39.5)	57 (45.2)	
**LGA, *N* (%)**	4 (1.9)	2 (2.3)	2 (1.6)	
**Length at birth (cm), mean (SD)**	33.5 (2.7)	33.4 (2.5)	33.6 (2.9)	0.66
**Head circumference at birth (cm), mean (SD)**	24.1 (2.0)	24.2 (1.8)	24.1 (2.3)	0.71
**Non-invasive ventilation (days), median [IQR]**	42 [28–60]	40 [28–57]	42 [28–61]	0.41
**Invasive ventilation (days), median [IQR]**	13 [5–29]	13 [5–31]	12 [5–29]	0.39
**Surgical gastrointestinal disease, *N* (%)**	23 (10.8)	8 (9.3)	15 (11.9)	0.55
**Severe brain disease, *N* (%)**	34 (16.0)	15 (17.4)	19 (15.1)	0.64
**Infections, *N* (%)**	94 (44.3)	36 (41.9)	58 (46.0)	0.54
**Bronchopulmonary dysplasia, *N* (%)**	96 (45.3)	39 (45.3)	57 (45.2)	0.98
**Retinopathy of prematurity stage 3–4, *N* (%)**	34 (16.0)	13 (15.1)	21 (16.7)	0.82
**Feeding at discharge, *N* (%)**				
Exclusive human milk feeding	29 (13.7)	8 (9.3)	21 (16.7)	0.36
Mixed feeding (human milk and formula milk)	35 (16.5)	17 (19.7)	18 (14.3)	
Exclusive formula feeding	148 (69.8)	61 (70.9)	87 (69.0)	
**Weight at discharge (g), mean (SD)**	2942 (1026.3)	2957 (1000)	2932 (992)	0.83
**Length of hospital stay (days), median [IQR]**	90 [70–130]	87 [70–128]	98 [73–132]	0.39

**Fig. 2:**
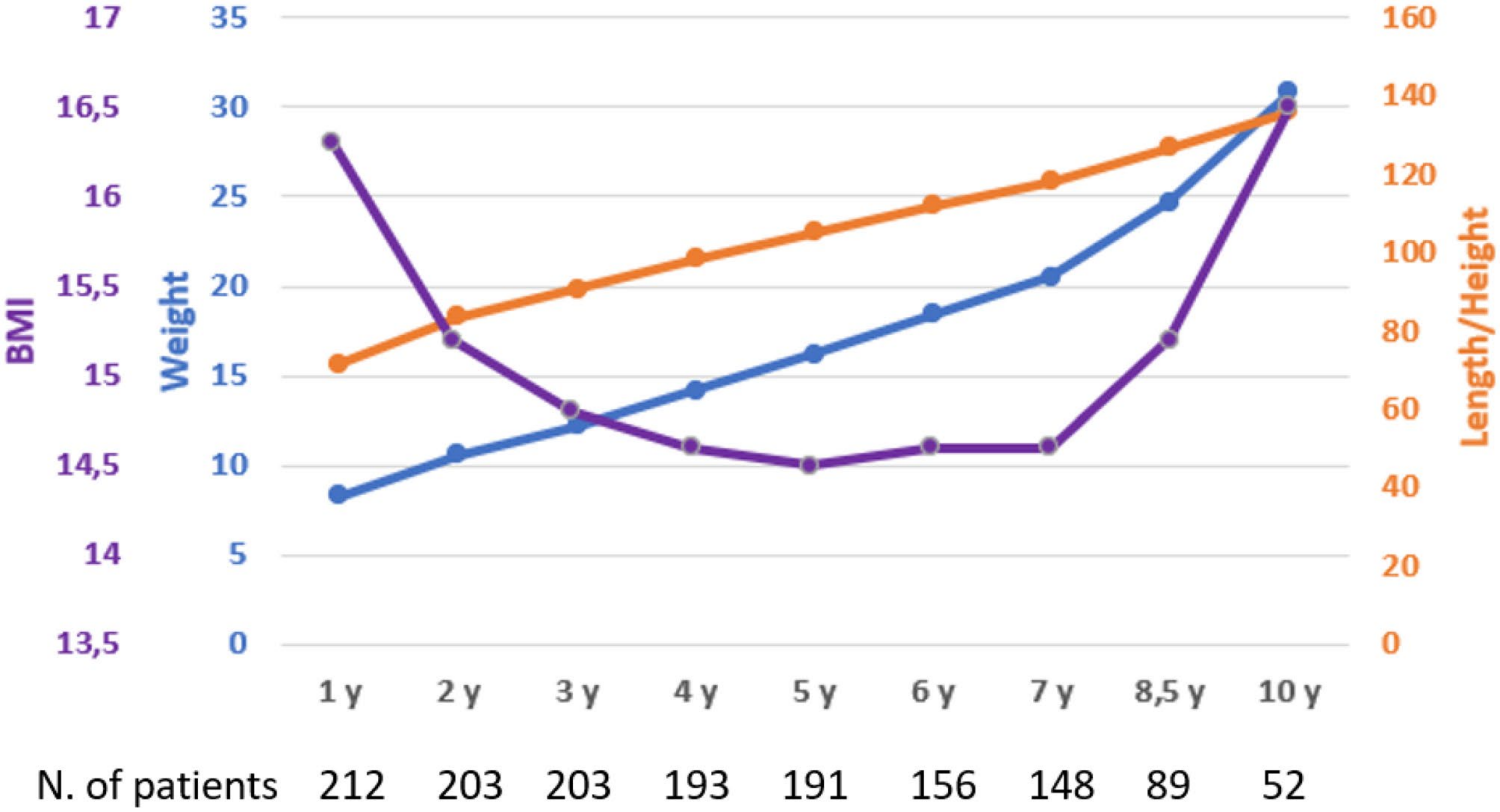
Multiple line graph representation of weight, length/height, and BMI trajectories of the study population throughout the study period

Trajectories of weight, length/height, and BMI are graphically represented in Fig. 2. As for head circumference, mean values measured during each follow-up visit in the first 3 years were 44.6 (± 1.9), 46.9 (± 1.9), and 47.7 (± 1.7), respectively.

Regarding growth trajectories, 43% and 32% of children still showed weight values lower than the 10th centile at 5 and 7 years of age, respectively. Moreover, 32% and 21% of them presented length values lower than the 10th centile at 5 and 7 years, respectively (Suppl. Fig. 1).

The 40.6% of the study population presented EAR, and the 21.5% showed it before 4 years of age.

At multivariate binary logistic regression analysis only exclusive or partial formula milk feeding was independently associated with a higher risk of developing EAR compared to exclusive human milk feeding (Table 2).

**Table 2:** Factors associated with early adiposity rebound at multivariate binary logistic regression analysis

Variable	Early adiposity rebound		
	**OR**	**95% C.I.**	***p***
**Pregnancy**			
Single vs. multiple	0.8	0.4–1.6	0.75
**Mode of delivery**			
Vaginal vs. caesarean section	1.1	0.4–3.0	0.82
**Gender**			
Male vs. female	0.9	0.5–1.8	0.98
**Birth centile**			
SGA vs. AGA/LGA	0.8	0.4–1.6	0.64
**Feeding at discharge**			
Complementary/exclusive formula feeding vs. exclusive human milk feeding	3.7	1.1–11.9	0.03
**Maternal education**			
No University degree vs. University degree	1.2	0.9–1.4	0.09

**Fig. 3:**
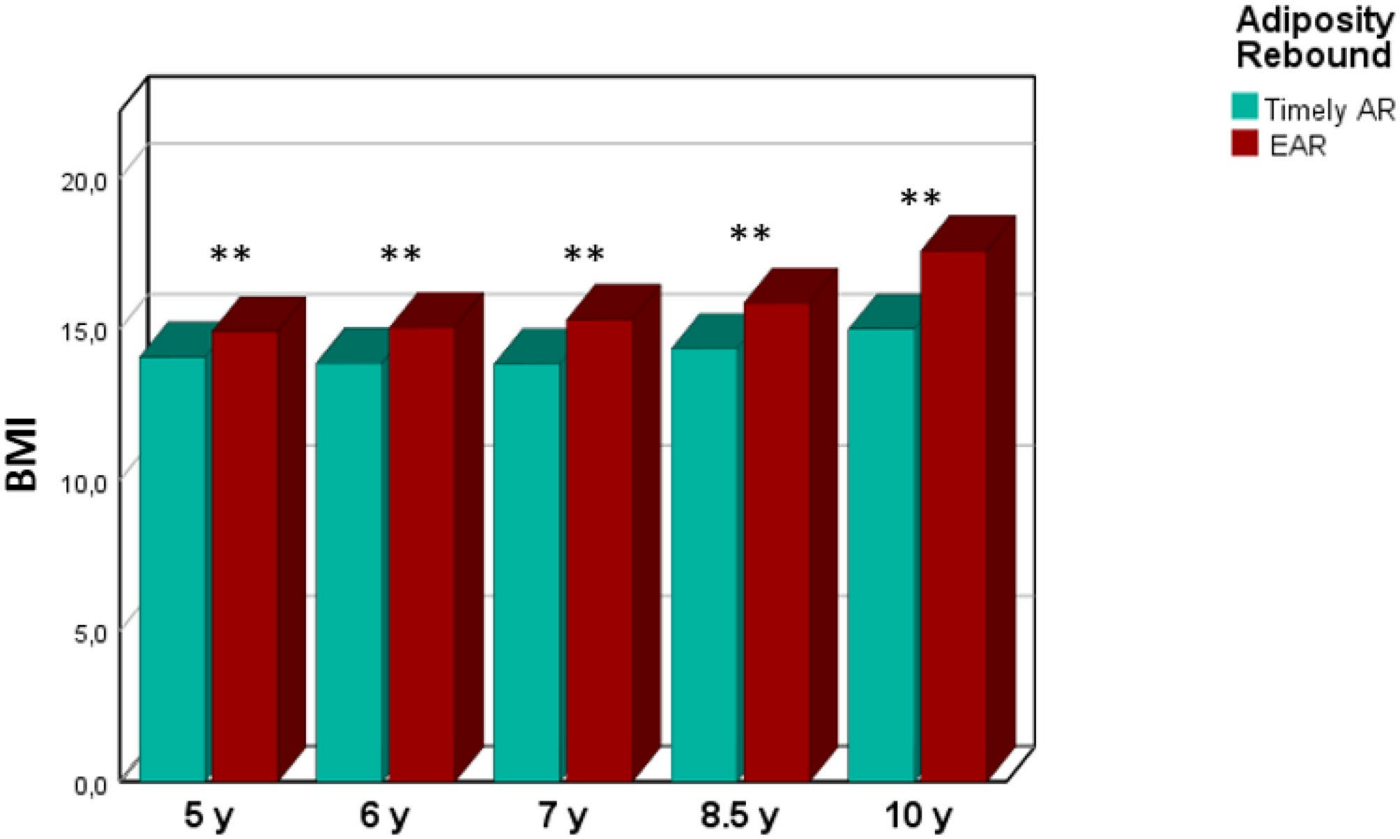
Mean BMI values in children with early adiposity rebound (EAR) and timely adiposity rebound (AR). **p < 0.001. C.I. 95% 5 years, − 1.3; − 0.4. C.I. 95% 6 years, − 1.9; − 0.9. C.I. 95% 7 years, − 2.2; − 1.0. C.I. 95% 8.5 years, − 2.8; − 1.1. C.I. 95% 10 years, − 4.6; − 1.9

Mean BMI of children with EAR was significantly higher than that of children without EAR at 5, 6, 7, 8.5, and 10 years (Fig. 3).

Furthermore, the prevalence of overweight and obesity among children with EAR was higher than that in the timely AR group at 6 (10% vs. 1%; 5% vs. 1%, respectively, *p* = 0.009) and 7 years (12.5% vs. 1%; 7% vs. 1%, respectively, *p* = 0.001). At 10 years, such difference in the rate of overweight and obesity persisted, with 21% of children with EAR being overweight and 16.7% of them obese vs. 3% of children being overweight and no one obese in the timely AR group (*p* = 0.009).

**Fig. 4:**
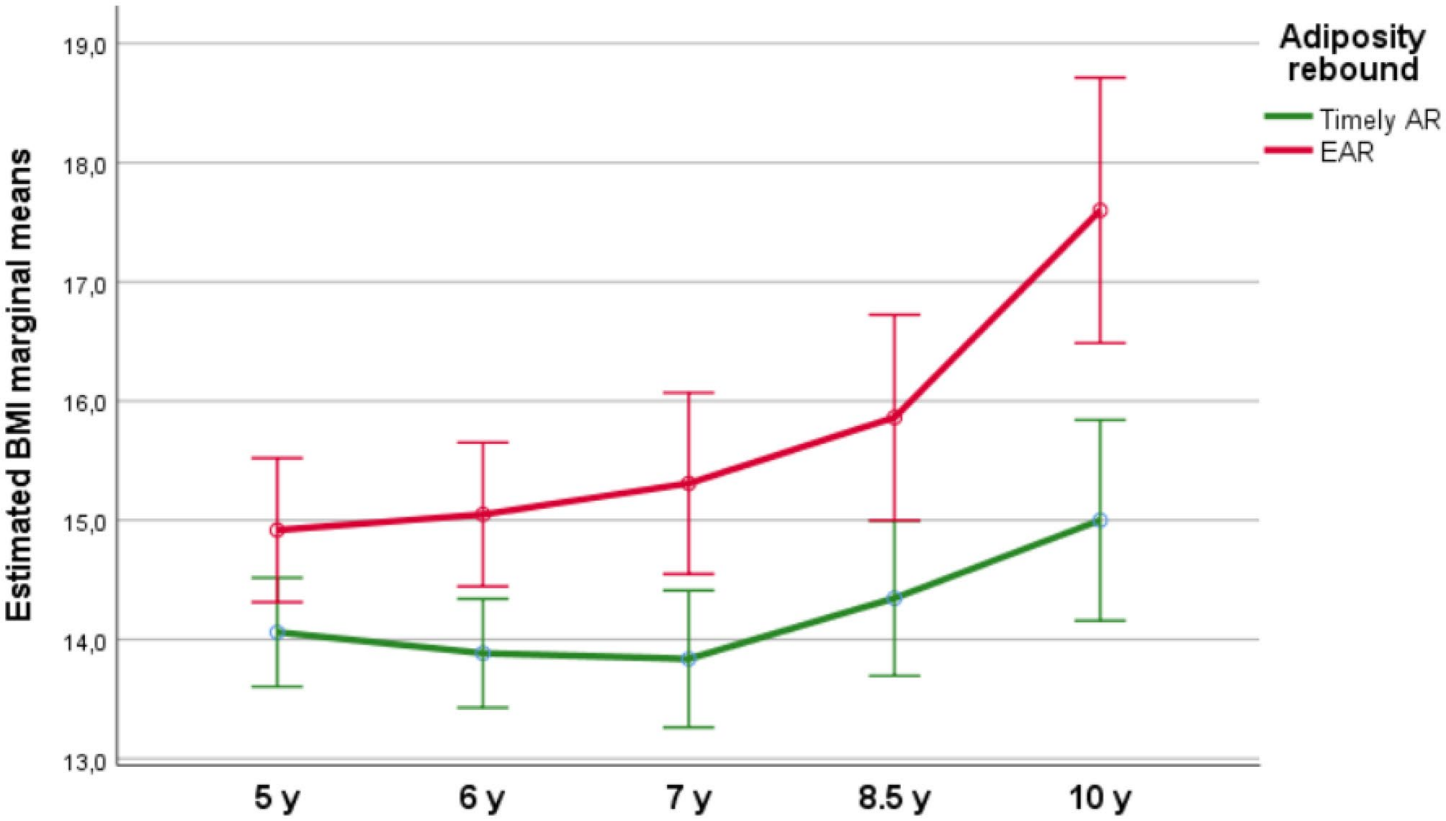
Estimated marginal means of BMI with standard errors at different time points showing the effect of EAR on BMI overtime. A Repeated Measures ANOVA showed a significant effect of EAR on BMI (*F*(2.03) = 5.53, *p* = 0.006, partial *η*2 = 0.15)

A Repeated Measures ANOVA was performed to evaluate the effect of EAR on BMI measured at different time points. Mauchly’s test indicated that the assumption of sphericity had been violated, *χ*2 = 64.4, *p* < 0.001, and therefore degrees of freedom were corrected using Greenhouse–Geisser estimates of sphericity (*ε* = 0.51). The effect of EAR on BMI through time was significant at the 0.05 level, *F*(2.03) = 5.53, *p* = 0.006, partial *η*2 = 0.15. Post hoc test identified a significant effect of EAR on BMI at each time point (all *p* < 0.05) (Fig. 4).

Mean SP values of children with EAR were significantly higher at 4 years of age than those of children with timely AR (101.58 ± 8.0 vs. 95.8 ± 6.7 mmHg, *p* < 0.05). Conversely, no other significant difference was found among the two groups in terms of SP at the following ages and DP at any time during the study period.

## Discussion

To our knowledge, this is the first study investigating the prevalence of EAR in a cohort of ex-preterm ELBW children. In our cohort, the prevalence of EAR was higher than that reported in the general population [9, 27, 28], although the lack of case–control studies comparing term and preterm infants make it difficult to draw a definite conclusion on the impact of prematurity and birth weight on the timing of AR.

The prevalence of EAR found in the present study is in line with that reported in preterm infants by other Authors. Baldassarre et al. [11] conducted a prospective study including 100 children born prematurely and reported that 54% of them showed EAR. In said study, ELBW infants represented the 1% of the study population (and the 2% of the EAR group). Children who presented EAR showed higher values of BMI at 7 years of age, whereas the prevalence of obesity or overweight was not different in children with early or timely AR. Contrary to this latter finding, our study revealed a notable association between EAR and an increased prevalence of overweight and obesity at 6, 7, and 10 years. The data indicate that children experiencing EAR are at a substantially elevated risk of developing overweight and obesity, and this disparity persists over time. To further investigate the impact of EAR on BMI across different age points, a Repeated Measures ANOVA was conducted. The analysis revealed a significant effect of EAR on BMI over time, suggesting that EAR contributes to a lasting influence on BMI trajectories. These findings contribute valuable insights into the potential long-term consequences of EAR on growth outcomes in ELBW infants.

The discrepancy between our findings and Baldassarre’s could be partially explained because of the different populations considered: in the paper by Baldassarre et al. the enrolled population comprised children born at a mean gestational age of 34 (± 2) weeks, mainly with a normal birth weight, whereas we focused on children born prematurely with ELBW. Also consistently with our findings, Nakayama et al. [12] reported that 38% of the very low birth weight infants (VLBW; i.e., birth weight < 1500 g) included in the analysis had EAR. The 60.5% of the infants included in the EAR group were ELBW, although the occurrence of EAR was not found to be associated with birth weight. At 7 years of age, children with EAR had higher BMI values than those with timely AR. Specifically, the 14.3% in the EAR group were obese, whereas children in the timely AR group had a normal weight.

In the present study, children with EAR showed higher SP values at 4 years of age than those in the timely AR group. Conversely, no difference in either the values of SP at any other study time point nor DP values throughout the follow-up period was found among the two groups. The higher SP in the EAR group at 4 years must be considered with caution since it was no longer found at the following study points. We could speculate that the evidence of isolated high SP values could be at least partially ascribed to white coat effect-related anxiety during the measurements [29]. However, several authors have pointed out that low birth weight preterm-born adolescents and young adults have higher blood pressure values than normal birth weight term-born controls [30, 31]. Monitoring blood pressure changes in preterm-born children may therefore prove useful in detecting subjects at risk of developing hypertension later in life.

According to the findings of the present study, being discharged from the NICU as exclusively or partially formula fed increases the risk of developing EAR. This finding is not surprising, considering the well-known role of mode of feeding in modulating early life growth pattern [32–34]. Indeed, Briollais et al. [35] have identified the genes and the related epigenetic modifications associated with the strict relationship between breastfeeding and growth pattern, further highlighting the importance of breastfeeding in reducing the risk of overweight and obesity. Moreover, human milk feeding of preterm infants during the early postnatal period has been associated with slower postnatal growth compared to formula feeding and with the recovery of body composition development in terms of promotion of lean mass deposition [36]. While Nakayama et al. did not consider mode of feeding as a possible confounder in their analysis, Baldassarre et al. results only showed a weak association between breastfeeding and timely AR [11, 12]. Conversely, a large population-based longitudinal study by Lin et al. [34] including term and preterm-born children showed that those who had been breastfed longer than 4 months had later AR than their counterparts who had not been breastfed or had been breastfed for shorter than 4 months. The association between breastfeeding and AR timing in preterm infants is still a matter of debate and further studies are needed to elucidate this important aspect.

In our cohort, nearly one-third of infants still presented weight values lower than the 10th centile at 7 years of age. This growth pattern, while indicating that catch-up growth could occur later in childhood [37], could also suggest a potential additional risk factor for the development of metabolic syndrome later in life. Indeed, the achievement of a catch-up growth within 12–18 months post-term has been reported to positively affect long-term outcomes, whereas faster weight gain during childhood seems to be associated with an increased risk of later metabolic syndrome and cardiovascular disease [38, 39]. Lastly, we could speculate that even if future, currently lacking, studies were to demonstrate that premature-born ELBW infants are not at higher risk of EAR than the general population, the sole known correlation of EAR with adverse metabolic outcomes [8–10] would justify monitoring the variations of BMI in this delicate population and the implementation of measures aimed at preventing its excessive and untimely increase. Indeed, EAR might be considered as an additional risk factor for an already at risk-population and every attempt at reducing such risk should be welcomed. The promotion of exclusive breastfeeding should therefore be recommended, also in light of the already well known beneficial effects on body composition and cardiovascular health [36, 40].

The present study was conducted on a relatively large cohort of ELBW infants followed up for a long period of time. However, the study has some limitations. Firstly, given the retrospective design, we analyzed data that were entered into a clinical database without an *ab initio* research purpose, that is following specific study requirements. Consequently, variables that may have an impact on the outcome considered may not have been recorded. Therefore, a bias from potential unmeasured confounders cannot be excluded. Secondly, despite efforts to address or account for misclassification during the study design or analysis, through careful study design and accurate data collection methods, some degree of bias may still persist. Moreover, although we established specific and precise inclusion and exclusion criteria, the potential presence of some residual selection bias in our study cannot be ruled out. Finally, the monocentric nature may limit the generalizability of our study findings. Acknowledging these limitations, we stress the provisional nature of our findings and the necessity for future research to corroborate and refine our understanding of possible predictors of EAR in preterm-born children.

## Conclusions

Preterm ELBW infants in our cohort developed EAR in a relatively high percentage of cases. In this already at-risk population, EAR may represent a further risk factor for adverse metabolic outcomes in addition to prematurity itself and its related complications. Monitoring preterm-born children’s growth, including BMI changes, within a long-term follow-up program, lasting at least up to school age, and promoting and supporting human milk feeding in this vulnerable population is advisable.

### Supplementary Information

Below is the link to the electronic supplementary material.

Supplementary file1 (DOCX 51 KB)

## Data Availability

The dataset used and analysed during the current study is available from the corresponding author on reasonable request.

## Abbreviations

AGA: Adequate for gestational age
AR: Adiposity rebound
BMI: Body mass index
BPD: Bronchopulmonary dysplasia
DP: Diastolic pressure
EAR: Early adiposity rebound
ELBW: Extremely low birth weight
LGA: Large for gestational age
NICU: Neonatal intensive care unit
ROP: Retinopathy of prematurity
SD: Standard deviations
SGA: Small for gestational age
SP: Systolic pressure
SPSS: Statistical package for the social sciences
VLBW: Very low birth weight

## References

[1] Vogel JP, Chawanpaiboon S, Moller A-B (2018). The global epidemiology of preterm birth. Best Pract Res Clin Obstet Gynaecol.

[2] Harrison MS, Goldenberg RL (2016). Global burden of prematurity. Semin Fetal Neonatal Med.

[3] Bell EF, Hintz SR, Hansen NI (2022). Mortality, in-hospital morbidity, care practices, and 2-year outcomes for extremely preterm infants in the US, 2013–2018. JAMA - J Am Med Assoc.

[4] Crump C (2020). An overview of adult health outcomes after preterm birth. Early Hum Dev.

[5] Luu TM, Katz SL, Leeson P (2016). Preterm birth: risk factor for early-onset chronic diseases. CMAJ Can Med Assoc J.

[6] Simeoni U, Armengaud J-B, Siddeek B, Tolsa J-F (2018). Perinatal origins of adult disease. Neonatology.

[7] Mericq V, Martinez-Aguayo A, Uauy R (2017). Long-term metabolic risk among children born premature or small for gestational age. Nat Rev Endocrinol.

[8] Rolland-Cachera MF, Deheeger M, Maillot M (2005). Bellisle F (2006) Early adiposity rebound: causes and consequences for obesity in children and adults. Int J Obes.

[9] Hughes AR, Sherriff A, Ness AR, Reilly JJ (2014). Timing of adiposity rebound and adiposity in adolescence. Pediatrics.

[10] Koyama S, Ichikawa G, Kojima M (2014). Adiposity rebound and the development of metabolic syndrome. Pediatrics.

[11] Baldassarre ME, Di Mauro A, Caroli M (2020). Premature birth is an independent risk factor for early adiposity rebound: longitudinal analysis of BMI data from birth to 7 years. Nutrients.

[12] Nakayama K, Ichikawa G, Naganuma J (2022). Adiposity rebound in very-low-birth-weight infants. J Pediatr Endocrinol Metab JPEM.

[13] Papageorghiou AT, Kennedy SH, Salomon LJ (2018). The INTERGROWTH-21st fetal growth standards: toward the global integration of pregnancy and pediatric care. Am J Obstet Gynecol.

[14] Jobe AH, Bancalari E (2001). Bronchopulmonary dysplasia. Am J Respir Crit Care Med.

[15] Molinari A, Weaver D, Jalali S (2017). Classifying retinopathy of prematurity. Community Eye Health.

[16] Agostoni C, Grandi F, Gianni M (1999). Growth patterns of breast fed and formula fed infants in the first 12 months of life: an Italian study. Arch Dis Child.

[17] Kuczmarski RJ, Ogden CL, Guo SS (2002). 2000 CDC Growth Charts for the United States: methods and development. Vital Health Stat.

[18] Cacciari E, Milani S, Balsamo A (2002). Italian cross-sectional growth charts for height, weight and BMI (6–20 y). Eur J Clin Nutr.

[19] de Onis M, Garza C, Victora CG (2004). The WHO Multicentre Growth Reference Study: planning, study design, and methodology. Food Nutr Bull.

[20] Vizzari G, Morniroli D, Tiraferri V (2022). Postnatal growth of small for gestational age late preterm infants: determinants of catch-up growth. Pediatr Res.

[21] de Onis M, Onyango AW, Borghi E (2007). Development of a WHO growth reference for school-aged children and adolescents. Bull World Health Organ.

[22] Bramsved R, Regber S, Novak D (2018). Parental education and family income affect birthweight, early longitudinal growth and body mass index development differently. Acta Paediatr.

[23] Yuan C, Gaskins AJ, Blaine AI (2016). Association between cesarean birth and risk of obesity in offspring in childhood, adolescence, and early adulthood. JAMA Pediatr.

[24] Kanter R, Caballero B (2012). Global gender disparities in obesity: a review. Adv Nutr Bethesda Md.

[25] Dulloo AG, Jacquet J, Seydoux J, Montani J-P (2006). The thrifty ‘catch-up fat’ phenotype: its impact on insulin sensitivity during growth trajectories to obesity and metabolic syndrome. Int J Obes.

[26] Rito AI, Buoncristiano M, Spinelli A (2019). Association between characteristics at birth, breastfeeding and obesity in 22 countries: the WHO European childhood obesity surveillance initiative – COSI 2015/2017. Obes Facts.

[27] Rolland-Cachera MF, Deheeger M, Bellisle F (1984). Adiposity rebound in children: a simple indicator for predicting obesity. Am J Clin Nutr.

[28] Zhou J, Zhang F, Qin X (2022). Age at adiposity rebound and the relevance for obesity: a systematic review and meta-analysis. Int J Obes.

[29] Matsuoka S, Kawamura K, Honda M, Awazu M (2002). White coat effect and white coat hypertension in pediatric patients. Pediatr Nephrol Berl Ger.

[30] Doyle LW, Faber B, Callanan C, Morley R (2003). Blood pressure in late adolescence and very low birth weight. Pediatrics.

[31] Keijzer-Veen MG, Dülger A, Dekker FW (2010). Very preterm birth is a risk factor for increased systolic blood pressure at a young adult age. Pediatr Nephrol Berl Ger.

[32] Boquien C-Y (2018). Human milk: an ideal food for nutrition of preterm newborn. Front Pediatr.

[33] Bardanzellu F, Peroni DG, Fanos V (2020). Human breast milk: bioactive components, from stem cells to health outcomes. Curr Nutr Rep.

[34] Lin D, Chen D, Huang J (2021). Pre-birth and early-life factors associated with the timing of adiposity peak and rebound: a large population-based longitudinal study. Front Pediatr.

[35] Briollais L, Rustand D, Allard C (2021). DNA methylation mediates the association between breastfeeding and early-life growth trajectories. Clin Epigenetics.

[36] Cerasani J, Ceroni F, De Cosmi V (2020). Human milk feeding and preterm infants’ growth and body composition: a literature review. Nutrients.

[37] Ferguson EC, Wright NP, Gibson AT (2017). Adult height of preterm infants: a longitudinal cohort study. Arch Dis Child.

[38] Embleton ND, Korada M, Wood CL (2016). Catch-up growth and metabolic outcomes in adolescents born preterm. Arch Dis Child.

[39] Raaijmakers A, Jacobs L, Rayyan M (2017). Catch–up growth in the first two years of life in Extremely Low Birth Weight (ELBW) infants is associated with lower body fat in young adolescence. PLoS ONE.

[40] El-Khuffash A, Lewandowski AJ, Jain A (2021). Cardiac performance in the first year of age among preterm infants fed maternal breast milk. JAMA Netw Open.

