# Supplementary figures and images for: Exclusive human milk feeding and prevalence of early adiposity rebound in ELBW infants: a retrospective cohort study
Source: Eur J Pediatr. 2023 Dec 19;183(3):1295–303. doi: 10.1007/s00431-023-05374-6 (PMC10950974; doi:10.1007/s00431-023-05374-6)

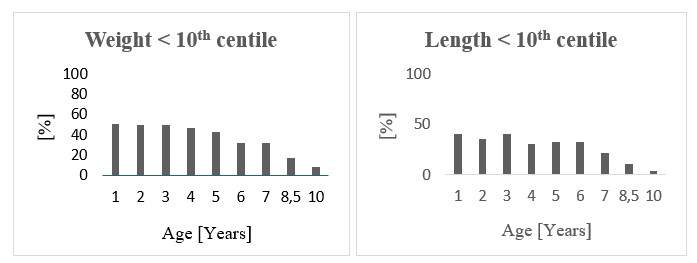


**Suppl. Fig. 1.** Percentage of infants with weight and length < 10th centile throughout the study period.

Supplement: Supplementary file 1 — Supplementary file1 (DOCX 51 KB) [file 431_2023_5374_MOESM1_ESM.docx]
